# Integrated transcriptomic and proteomic profiling reveals C3-associated hippocampal neuroimmune remodeling in sodium bicarbonate-treated sepsis-associated encephalopathy

**DOI:** 10.3389/fnins.2026.1954272

**Published:** 2026-10-07

**Authors:** Housheng Deng, Hua Ling, Lie Liu, Qiuli He, Hao Wang, Zhiming Zhang

**Affiliations:** 1 The First People's Hospital of Chenzhou, Medical Education Center of Jinan University, Chenzhou, China; 2 The Affiliated Suzhou Hospital of Nanjing Medical University, Nanjing, China; 3 The Second Affiliated Hospital of Nanjing Medical University, Nanjing, China; 4 The First Affiliated Hospital of Jinan University, Guangzhou, China

**Keywords:** complement component 3, neuroinflammation, reactive astrocytes, sepsis-associated encephalopathy, sodium bicarbonate

## Abstract

**Background:**

Sepsis-associated encephalopathy (SAE) is a neurological complication of sepsis characterized by cognitive impairment and persistent brain dysfunction. Although neuroinflammation and glial activation contribute to SAE pathogenesis, the molecular alterations underlying hippocampal neuroimmune dysfunction remain incompletely understood. The potential role of NaHCO₃ in SAE-associated neurological dysfunction remains unclear.

**Methods:**

An LPS-induced mouse model of SAE was established to investigate the effects of NaHCO₃ administration. Survival and cognitive function were evaluated using survival analysis, novel object recognition, open-field, and Y-maze tests. Hippocampal transcriptomic and proteomic profiling was performed, followed by cross-omics integration. Candidate molecules were further assessed by qRT-PCR, Western blotting, and immunofluorescence.

**Results:**

NaHCO₃ treatment improved survival and alleviated cognitive deficits in SAE mice. Transcriptomic and proteomic profiling revealed extensive hippocampal molecular alterations, with functional enrichment highlighting immune- and inflammation-related processes and pathways. Three-group cross-omics analysis identified 18 candidates meeting an FDR < 0.05 at both omics levels and showing a concordant SAE-associated increase followed by a decrease after NaHCO₃ treatment. Among these candidates, C3 was prioritized based on its cross-omics expression pattern and neuroimmune relevance. qRT-PCR and Western blot analyses supported increased hippocampal C3 expression during SAE and its responsiveness to NaHCO₃ treatment. Immunofluorescence analysis revealed increased spatial association between C3 and GFAP-positive astrocytic structures during SAE, which was attenuated following NaHCO₃ treatment.

**Conclusion:**

NaHCO₃ treatment was associated with improved neurological outcomes and altered hippocampal molecular profiles in LPS-induced SAE. Cross-omics analysis identified C3 among candidate molecules associated with SAE and NaHCO₃ responsiveness. The increased C3–GFAP spatial association suggests that complement-associated alterations accompany astrocytic responses during SAE. The causal role of C3 and the mechanisms underlying the effects of NaHCO₃ require further investigation.

## 1 Introduction

Sepsis-associated encephalopathy (SAE) is a common and severe neurological complication of systemic sepsis, characterized by diffuse brain dysfunction, cognitive impairment, and long-term neurological consequences among survivors (Gofton and Young, 2012; Mazeraud et al., 2020). Despite improvements in intensive care management, SAE remains associated with substantial morbidity and mortality, highlighting the urgent need to better understand the mechanisms underlying sepsis-induced brain injury. Although peripheral inflammation is recognized as a major contributor to SAE, how systemic immune disturbances translate into persistent dysfunction within vulnerable brain regions, particularly the hippocampus, remains incompletely understood (Prescott and Angus, 2018).

Neuroinflammation has emerged as a central mechanism involved in SAE pathogenesis (Semmler et al., 2008). Systemic inflammatory activation during sepsis can disrupt blood–brain barrier integrity, alter cerebral homeostasis, and induce complex interactions among immune cells, glial populations, and neurons (Sofroniew, 2015; Fisher and Liddelow, 2024). Within the central nervous system, microglia and astrocytes serve as key regulators of neuroimmune communication and neuronal function under pathological conditions (Cheng et al., 2026). Increasing evidence indicates that reactive astrocytes actively participate in inflammatory responses and synaptic regulation through alterations in gene expression and the production of immune-related molecules, suggesting an important role for astrocyte-mediated mechanisms in sepsis-associated cognitive dysfunction (Liddelow et al., 2017; Escartin et al., 2021; Ogunleye et al., 2026).

The complement system, traditionally considered a component of innate immunity, has recently been recognized as an important regulator of neuroimmune interactions (Tian et al., 2025; Wang L. et al., 2026). Complement component 3 (C3), a central molecule within the complement cascade, is markedly induced under various inflammatory conditions and is associated with specific reactive astrocytic states (Ricklin et al., 2010; Hong et al., 2016; Mou et al., 2022). Previous studies have demonstrated that complement activation contributes to synaptic remodeling, neuroinflammation, and neuronal dysfunction in multiple neurological disorders (Hong et al., 2016). However, whether hippocampal C3-associated astrocytic responses contribute to SAE-related cognitive dysfunction remains unclear.

Sodium bicarbonate (NaHCO₃) is a clinically available alkalinizing agent widely used for the management of metabolic disturbances (Mohebbi et al., 2023; Prescott et al., 2026). Beyond its established effects on systemic acid–base homeostasis, experimental observations have suggested that changes in bicarbonate availability and extracellular pH may be accompanied by alterations in cellular metabolism and inflammatory responses (O'neill et al., 2016; Bastola et al., 2024; Wang D. et al., 2026). However, the potential effects and molecular mechanisms of NaHCO₃ in sepsis-associated neurological dysfunction remain poorly understood. In particular, whether NaHCO₃ affects complement-associated astrocytic responses during SAE has not been investigated.

In the present study, we established an LPS-induced SAE mouse model and employed integrated transcriptomic and proteomic approaches to characterize hippocampal molecular alterations associated with SAE and NaHCO₃ intervention. We identified C3 as a candidate neuroimmune-associated molecule and validated its expression changes at both transcriptional and protein levels. Furthermore, immunofluorescence analysis was performed to examine the spatial association between C3 and GFAP-positive astrocytic signals in the hippocampus. Our findings provide new insights into hippocampal neuroimmune remodeling during SAE and identify C3 as a candidate molecule for further mechanistic investigation.

## 2 Materials and methods

### 2.1 Animals and experimental design

Forty-eight specific pathogen-free (SPF) male C57BL/6 J mice (8 weeks of age, 20–22 g) were obtained from Beijing Vital River Laboratory Animal Technology Co., Ltd. (Beijing, China). Animals were housed under standard laboratory conditions (22 ± 2 °C, 50–60% humidity, 12-h light/dark cycle) with ad libitum access to irradiated chow and acidified drinking water. All mice were allowed to acclimatize for 1 week before experimentation.

All experimental procedures were approved by the Institutional Animal Care and Use Committee and were conducted in accordance with the National Institutes of Health Guide for the Care and Use of Laboratory Animals and the ARRIVE 2.0 guidelines. Every effort was made to minimize animal suffering and to reduce the number of animals used.

Following acclimation, mice were randomly assigned to three experimental groups (n = 16 per group) using a computer-generated randomization schedule: (1) Sham group, receiving intraperitoneal injection of sterile saline (10 mL/kg); (2) SAE group, receiving lipopolysaccharide (LPS; *Escherichia coli* O111: B4, Sigma-Aldrich, L4130, USA) at 5 mg/kg by intraperitoneal injection to induce sepsis-associated encephalopathy; and (3) NaHCO₃ treatment group, receiving LPS followed by sodium bicarbonate (NaHCO₃, 0.1 g/kg/day, intraperitoneally) for seven consecutive days. The NaHCO₃ dosage and treatment regimen were selected according to previous studies and our preliminary experiments demonstrating favorable tolerability and therapeutic efficacy.

LPS was administered between 08:00 and 09:00 to minimize circadian variation. Body weight, rectal temperature, and clinical severity scores were recorded every 12 h throughout the observation period. Survival was monitored daily for seven consecutive days. Behavioral assessments were performed after stabilization of systemic illness between days 5 and 7, followed by tissue collection for molecular and histological analyses. Investigators responsible for behavioral testing, image acquisition, and quantitative data analysis were blinded to treatment allocation throughout the study.

Of the 16 mice initially assigned to each group, 12 mice per group were prospectively designated for the 7-day Kaplan–Meier survival analysis, whereas the remaining 4 mice per group were not included in the survival-curve calculation. All animals were otherwise followed throughout the experimental period. At the time of behavioral assessment, 8 mice remained alive in the SAE group, including 5 mice from the survival-analysis cohort and 3 mice from the remaining 4 animals. All 8 surviving SAE mice were therefore included in the behavioral assessments. In the Sham and SAE + NaHCO₃ groups, in which more than 8 animals remained alive, 8 surviving mice per group were selected for behavioral testing to maintain an equal sample size across groups. The same behavioral cohort underwent the NOR, OFT, and Y-maze tests. Following behavioral assessment, hippocampal tissues from 4 mice per group were used for transcriptomic profiling, quantitative proteomics, qRT-PCR, and Western blotting, whereas tissues from the remaining 4 mice per group were used for immunofluorescence analysis.

### 2.2 Neurobehavioral assessments

Behavioral assessments were performed using the same cohort of 8 mice per group (*n* = 8 biological replicates/group). All animals in this cohort underwent the novel object recognition, open-field, and Y-maze tests. Behavioral experiments were conducted between 08:00 and 14:00 under controlled environmental conditions. Mice were acclimated to the behavioral testing room for at least 30 min before each experiment. All apparatuses were cleaned with 75% ethanol between trials to eliminate olfactory cues. Behavioral analyses were performed by investigators blinded to experimental grouping.

#### 2.2.1 Novel object recognition test

The novel object recognition (NOR) test was performed to evaluate recognition memory. Mice were habituated to the testing arena for 1 day, followed by a training session with two identical objects. During the test session conducted 2 h later, one familiar object was replaced with a novel object. Exploration behavior was automatically recorded using the ANY-maze tracking system (Stoelting, USA) and verified manually by an investigator blinded to group allocation. Recognition memory was expressed as the recognition index (RI), calculated as the time spent exploring the novel object divided by the total exploration time.

#### 2.2.2 Open field test

The open field test (OFT) was used to evaluate spontaneous locomotor activity and anxiety-like behavior. Each mouse was individually placed in the center of the apparatus and allowed to explore freely for 5 min. Total travel distance, average velocity, time spent in the central zone, and the number of center entries were automatically analyzed using the ANY-maze software.

#### 2.2.3 Y-maze spontaneous alternation test

Working memory was assessed using the spontaneous alternation Y-maze test. Mice were allowed to freely explore all three arms of the maze for 8 min. Arm entries and spontaneous alternations were automatically recorded. The spontaneous alternation percentage was calculated as the number of successful alternations divided by the total number of possible alternations [(total arm entries − 2)] × 100%.

### 2.3 Transcriptomic analysis

Transcriptomic analysis was performed using hippocampal tissues from 4 animals per group (*n* = 4 biological replicates/group). Total RNA was extracted from hippocampal tissues, and RNA concentration and purity were assessed using a NanoDrop 2000 spectrophotometer. RNA integrity was evaluated by agarose gel electrophoresis and an Agilent 5,300 system. Samples meeting the quality requirements (total RNA ≥ 1 μg, concentration ≥ 30 ng/μL, RQN > 6.5, and OD260/280 between 1.8 and 2.2) were used for library preparation.

Poly(A)-containing mRNA was enriched from total RNA using oligo (dT) magnetic beads and fragmented into approximately 300-bp fragments. First-strand cDNA was synthesized using random primers and reverse transcriptase, followed by second-strand cDNA synthesis with dUTP replacing dTTP. After end repair and A-tailing, sequencing adapters were ligated to the cDNA fragments. The dUTP-containing second-strand cDNA was subsequently degraded using uracil-DNA glycosylase (UDG), and the remaining cDNA was amplified by PCR. Following fragment selection and library purification, the libraries were subjected to high-throughput sequencing on the DNBSEQ-T7 platform.

Raw sequencing data underwent quality control, sequence alignment, and gene expression quantification before downstream differential-expression analysis. Differentially expressed genes (DEGs) between experimental groups were subsequently identified and used for functional enrichment and integrated transcriptomic–proteomic analyses.

### 2.4 Quantitative proteomic analysis

Quantitative proteomic analysis was performed using hippocampal tissues obtained from the same 4 animals per group used for transcriptomic profiling (*n* = 4 biological replicates/group). Hippocampal tissues were homogenized in lysis buffer containing protease inhibitors, and total protein concentrations were determined using the bicinchoninic acid (BCA) assay. Equal amounts of protein from each sample were subjected to tryptic digestion, followed by tandem mass tag (TMT)-based peptide labeling according to the manufacturer’s instructions.

Labeled peptides were fractionated by high-performance liquid chromatography (HPLC) and analyzed using high-resolution liquid chromatography–tandem mass spectrometry (LC–MS/MS). Raw mass spectrometry data were processed using MaxQuant software with peptide identification performed against the UniProt mouse protein database. Protein identification was accepted at a false discovery rate (FDR) of <1% at both the peptide and protein levels.

For pairwise comparisons, differentially expressed proteins (DEPs) were identified using Student’s t-test with a nominal *p* value < 0.05 and a fold-change threshold of >1.20 for upregulation or <0.83 for downregulation. Functional annotation and pathway enrichment analyses were subsequently performed using Gene Ontology (GO) and Kyoto Encyclopedia of Genes and Genomes (KEGG) databases.

### 2.5 Integrated transcriptomic–proteomic analysis

To characterize molecular changes shared between the transcriptomic and proteomic datasets, the two omics datasets were integrated based on corresponding gene symbols. For pairwise cross-omics analysis, differentially expressed genes (DEGs) and differentially expressed proteins (DEPs) identified in the SAE versus Sham and SAE + NaHCO₃ versus SAE comparisons were matched according to their corresponding gene symbols. Overlapping RNA–protein entries were identified, and the concordance or discordance of their expression directions was determined based on the corresponding fold changes. Pearson’s correlation analysis was performed to evaluate the relationship between transcriptomic and proteomic fold changes in each pairwise comparison.

To further identify candidate molecules exhibiting coordinated expression changes across the three experimental groups, one-way ANOVA was performed separately on the transcriptomic and proteomic datasets. Molecules with an FDR < 0.05 at both the transcriptomic and proteomic levels and showing a concordant three-group expression trajectory characterized by an SAE-associated increase followed by a decrease after NaHCO₃ treatment were retained as cross-omics candidates. Heatmaps were generated using normalized expression values to visualize their expression patterns across the Sham, SAE, and SAE + NaHCO₃ groups.

### 2.6 Quantitative real-time PCR

Quantitative RT-PCR analysis was performed using hippocampal samples from the same four animals per group used for transcriptomic and proteomic analyses (*n* = 4 biological replicates/group). Total RNA was isolated from hippocampal tissue using TRIzol reagent (Invitrogen, Carlsbad, CA, USA) according to the manufacturer’s instructions. RNA concentration and purity were determined spectrophotometrically, and complementary DNA (cDNA) was synthesized using a reverse transcription kit (Takara Bio, Shiga, Japan). Quantitative real-time PCR (qRT-PCR) was performed using SYBR Green Master Mix (Takara Bio, Japan) on a QuantStudio 5 Real-Time PCR System (Applied Biosystems, Foster City, CA, USA). The primer sequences were as follows: forward, 5′-GAGCGAAGAGACCATCGTACT-3′; reverse, 5′-TCTTTAGGAAGTCTTGCACAGTG-3′. GAPDH was used as the internal reference gene. Relative gene expression levels were calculated using the 2^-ΔΔCt method. All reactions were performed in technical triplicate, and the mean value of the three technical replicates was used to represent each biological sample for statistical analysis.

### 2.7 Western blot

Western blot analysis was performed using hippocampal samples from the same four animals per group used for transcriptomic, proteomic, and qRT-PCR analyses (n = 4 biological replicates/group). Tissues were homogenized in RIPA buffer (1 mM PMSF, 1% phosphatase inhibitor), centrifuged (12,000 g, 15 min, 4 °C), and supernatants were quantified by BCA. Proteins (20 μg/lane) were separated on 12% SDS-PAGE (80 V, 30 min; 120 V, 60 min) and transferred to 0.22 μm PVDF (100 V, 90 min). Membranes were blocked with 5% skim milk and incubated overnight at 4 °C with primary antibodies: C3 (rabbit polyclonal anti-C3, 1:1000, Abcam ab97462) and β-actin (1:5000, Proteintech 66,009). After TBST washes, HRP-conjugated secondary antibodies (1:5000) were applied, and immunoreactive bands were visualized using enhanced chemiluminescence (ECL) reagents. Band intensities were quantified using ImageJ software (National Institutes of Health, Bethesda, MD, USA). The C3-immunoreactive band used for densitometric analysis was located at approximately 187 kDa. Protein expression levels were normalized to β-actin and expressed relative to the Sham group.

### 2.8 Immunofluorescence staining and image analysis

Immunofluorescence analysis was performed using hippocampal sections from 4 animals per group (*n* = 4 biological replicates/group). Following transcardial perfusion with phosphate-buffered saline (PBS) and 4% paraformaldehyde, mouse brains were post-fixed, cryoprotected in 30% sucrose, and sectioned coronally at a thickness of 20 μm. Brain sections containing the hippocampus were permeabilized with 0.3% Triton X-100, blocked with 5% bovine serum albumin (BSA), and incubated overnight at 4 °C with primary antibodies against GFAP (rat polyclonal anti-GFAP, 1:500, Invitrogen 13–0300) and C3 (rabbit polyclonal anti-C3, 1:200, Abcam ab97462). After washing, sections were incubated with species-specific fluorescent secondary antibodies for 1 h at room temperature. Cell nuclei were counterstained with DAPI. Images were acquired using a laserscanning-confocal microscope (Leica Microsystems, Germany) under identical acquisition parameters for all experimental groups. Quantitative analyses were performed using ImageJ software with the JACoP plugin. Pearson’s correlation coefficient and Manders’ overlap coefficients (M1 and M2) were calculated to evaluate the spatial colocalization between GFAP and C3 signals. For quantitative colocalization analysis, three microscopic fields were analyzed for each animal, with four animals included per group (*n* = 4 biological replicates/group). Measurements obtained from the three fields of each animal were averaged to generate a single animal-level value, and each mouse was considered one biological replicate for statistical analysis.

### 2.9 Statistical analysis

All statistical analyses were performed using GraphPad Prism 9.0 (GraphPad Soft-ware, San Diego, CA, USA) and R software (version 4.3.0). Data are presented as mean ± standard deviation (SD) unless otherwise indicated. The normality of data distribution was assessed using the Shapiro–Wilk test, and homogeneity of variance was evaluated using Levene’s test prior to parametric analyses. Behavioral data (NOR, OFT, and Y-maze), qRT-PCR data, Western blotting data, and immunofluorescence measurements were compared among groups using one-way ANOVA followed by Tukey’s multiple-comparison test. Survival curves were generated using the Kaplan–Meier method and compared using the log-rank (Mantel–Cox) test.

For transcriptomic analysis, differential gene expression was analyzed using the DESeq2 package in R. Genes with a Benjamini–Hochberg-adjusted *p* value < 0.05 and |log₂ fold change| ≥ 1 were considered significantly differentially expressed. For pairwise proteomic comparisons, DEPs were identified using Student’s t-test with a nominal *p* value < 0.05 and a fold change >1.20 or <0.83.

For three-group candidate screening, one-way ANOVA was performed independently at the transcriptomic and proteomic levels, followed by Benjamini–Hochberg correction. Molecules with an FDR < 0.05 at both omics levels and satisfying the predefined cross-omics trajectory criteria were retained for subsequent candidate prioritization. Functional enrichment analyses, including Gene Ontology (GO) and Kyoto Encyclopedia of Genes and Genomes (KEGG) pathway analyses, were performed using the ClusterProfiler package, and adjusted *p* values were calculated using the Benjamini–Hochberg method. Correlation between transcriptomic and proteomic datasets was evaluated using Pearson’s correlation coefficient. Colocalization analyses of GFAP and C3 immunofluorescence signals were performed using Pearson’s correlation coefficient together with Manders’ overlap coefficients (M1 and M2). All statistical tests were two-tailed, and *p* < 0.05 was considered statistically significant. Statistical significance is indicated as *p < 0.05, **p < 0.01, ***p < 0.001, and ****p < 0.0001.

## 3 Results

### 3.1 Sodium bicarbonate alleviates survival impairment and cognitive dysfunction in LPS-induced SAE mice

Sepsis-associated encephalopathy (SAE) is characterized by acute neurological dysfunction and cognitive impairment. To evaluate the potential protective effects of sodium bicarbonate (NaHCO₃), an LPS-induced SAE mouse model was established, followed by NaHCO₃ administration (0.1 g/kg, i.p.) for 7 consecutive days.

SAE induction resulted in a significant reduction in survival compared with Sham animals, whereas NaHCO₃ treatment markedly improved survival probability during the observation period (Figure 1A).

**Figure 1:**
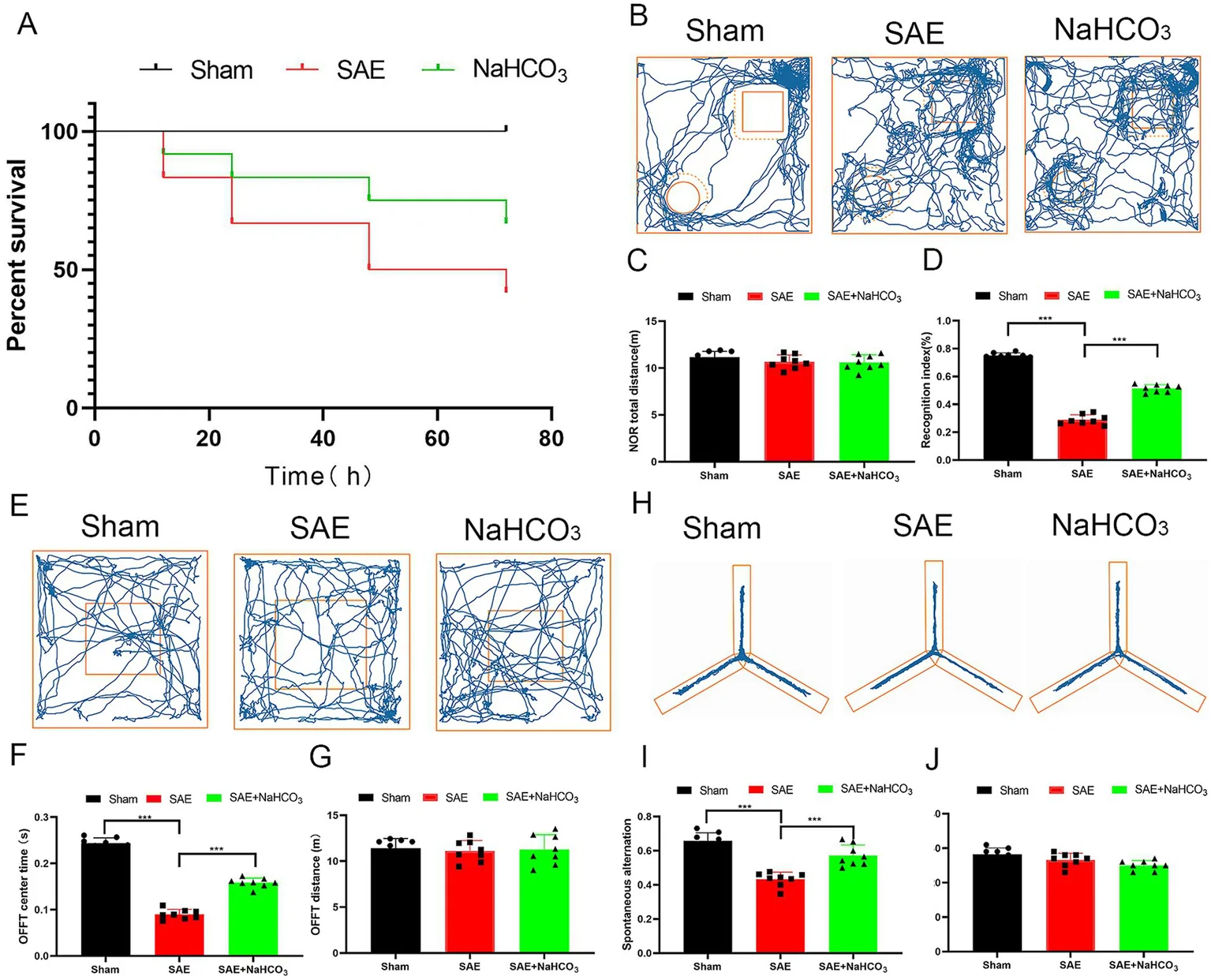
NaHCO₃ improves survival and cognitive function in mice with sepsis-associated encephalopathy (SAE). **(A)** Kaplan–Meier survival curves following LPS administration (*n* = 12 mice/group). NaHCO₃ (0.1 g/kg, intraperitoneally) was administered once daily for 7 consecutive days. **(B–D)** Novel object recognition test, including representative exploratory trajectories and quantitative assessment of recognition performance. **(E–G)** Open-field test, including representative locomotor trajectories and quantitative analyses of center-zone exploration and total distance traveled. **(H–J)** Y-maze test, including representative exploratory trajectories and quantitative analyses of spontaneous alternation and total arm entries. Behavioral analyses were performed using the same cohort of animals (*n* = 8 mice/group). Data are presented as mean ± SD. Survival curves were compared using the log-rank (Mantel–Cox) test. Behavioral data were analyzed using one-way ANOVA followed by Tukey’s multiple-comparison test. Statistical significance is indicated in the corresponding panels.

We next assessed SAE-associated cognitive impairment using behavioral paradigms. In the novel object recognition test, SAE mice exhibited impaired recognition memory, characterized by reduced discrimination of the novel object, while NaHCO₃ administration significantly improved cognitive performance (Figures 1B−D). Consistently, SAE mice displayed reduced exploratory activity and altered central zone exploration in the open field test, both of which were partially restored following NaHCO₃ treatment (Figures 1E−G). In the Y-maze test, SAE mice showed decreased spontaneous alternation behavior, indicating impaired working memory, whereas NaHCO₃ treatment significantly alleviated this deficit (Figures 1H−J).

Collectively, these findings indicate that NaHCO₃ administration ameliorates SAE- associated survival impairment and cognitive dysfunction.

### 3.2 Transcriptomic and proteomic profiling reveals hippocampal molecular alterations during SAE and following NaHCO₃ treatment

To characterize hippocampal molecular alterations associated with SAE and NaHCO₃ treatment, transcriptomic and proteomic profiling was performed using hippocampal tissues from the same four biological replicates per group. The overall experimental and sample-allocation scheme is shown in Supplementary Figure S1.

Transcriptomic analysis revealed distinct expression profiles among the three experimental groups. Principal component analysis (PCA) showed separation of the Sham, SAE, and SAE + NaHCO₃ groups, which was further supported by hierarchical clustering of differentially expressed genes (Figures 2A,B; Supplementary Table S1). Differential expression analysis identified 717 differentially expressed genes (DEGs) in SAE versus Sham, including 649 upregulated and 68 downregulated genes (Figure 2C; Supplementary Table S2). In the SAE + NaHCO₃ versus SAE comparison, 130 DEGs were identified, including 5 upregulated and 125 downregulated genes (Figure 2D; Supplementary Table S3).

**Figure 2:**
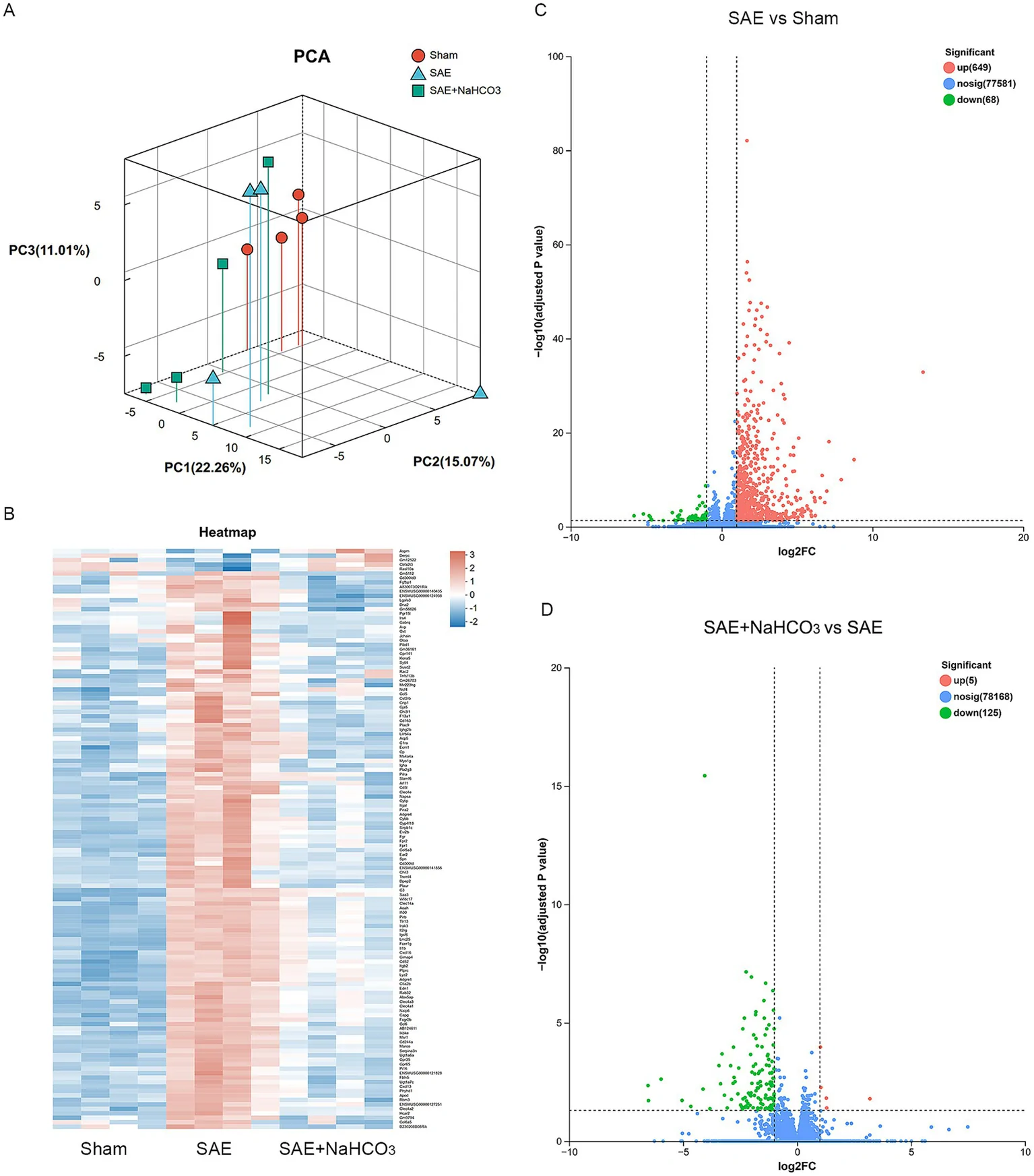
Transcriptomic profiling reveals hippocampal gene-expression alterations during SAE and following NaHCO₃ treatment. **(A)** Principal component analysis (PCA) of hippocampal transcriptomic profiles from the Sham, SAE, and SAE + NaHCO₃ groups (*n* = 4 biological replicates/group). **(B)** Hierarchical clustering heatmap showing the expression patterns of differentially expressed genes (DEGs) across the three experimental groups. **(C)** Volcano plot showing DEGs between the SAE and Sham groups. A total of 717 DEGs were identified, including 649 upregulated and 68 downregulated genes. **(D)** Volcano plot showing DEGs between the SAE + NaHCO₃ and SAE groups. A total of 130 DEGs were identified, including 5 upregulated and 125 downregulated genes. DEGs were identified using a Benjamini–Hochberg-adjusted *p* value < 0.05 and |log₂ fold change| ≥ 1. Red and blue indicate upregulated and downregulated genes, respectively.

Proteomic profiling similarly revealed group-dependent alterations in hippocampal protein expression. PCA and hierarchical clustering demonstrated distinct proteomic profiles among the three groups (Figures 3A,B; Supplementary Table S4). A total of 581 differentially expressed proteins (DEPs), including 429 upregulated and 152 downregulated proteins, were identified in SAE versus Sham (Figure 3C; Supplementary Table S5). Following NaHCO₃ treatment, 271 DEPs were identified relative to untreated SAE mice, including 78 upregulated and 193 downregulated proteins (Figure 3D; Supplementary Table S6).

**Figure 3:**
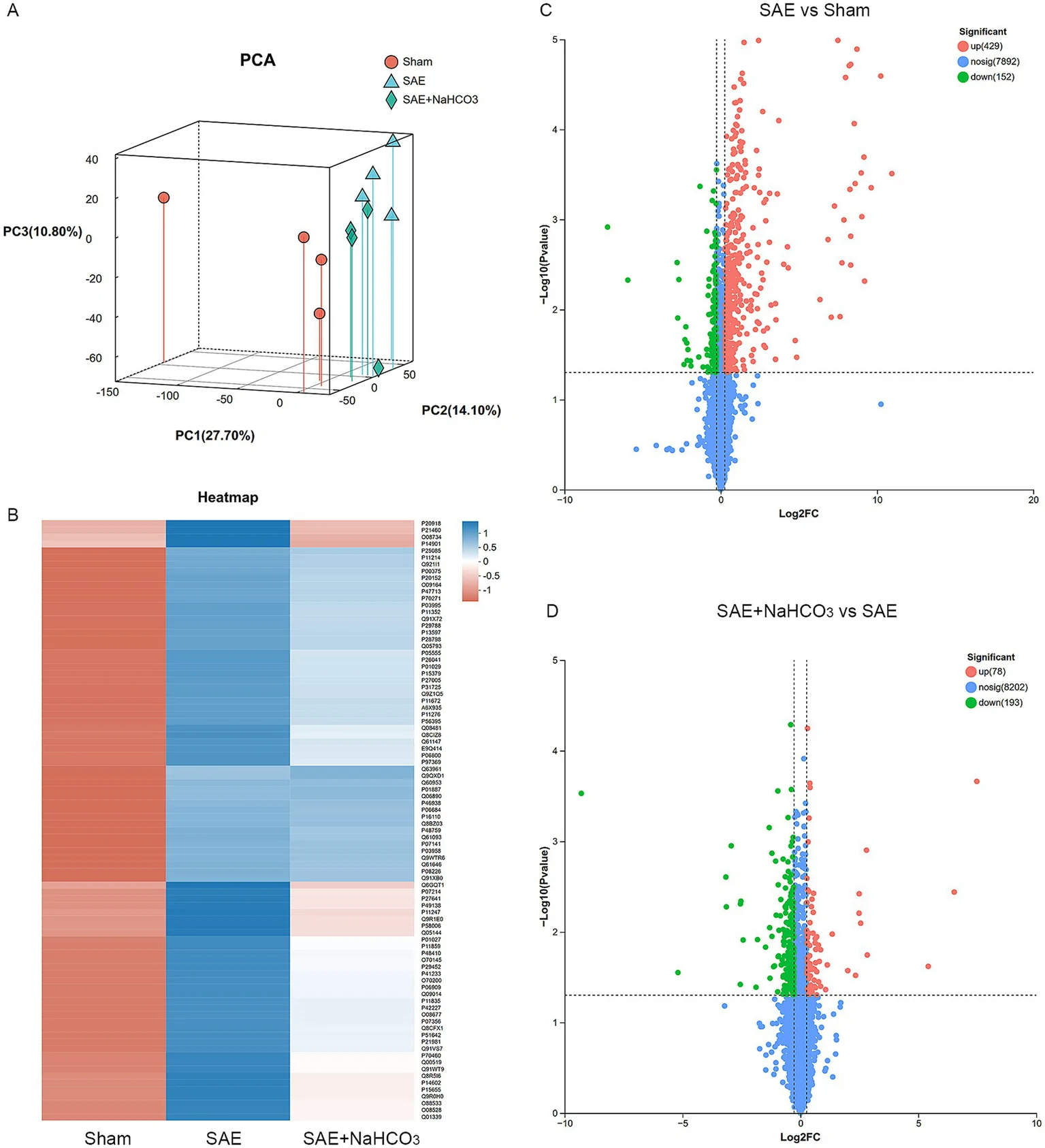
Quantitative proteomic profiling reveals hippocampal protein-expression alterations during SAE and following NaHCO₃ treatment. **(A)** Principal component analysis (PCA) of hippocampal proteomic profiles from the Sham, SAE, and SAE + NaHCO₃ groups (*n* = 4 biological replicates/group). **(B)** Hierarchical clustering heatmap showing the expression patterns of differentially expressed proteins (DEPs) across the three experimental groups. **(C)** Volcano plot showing DEPs between the SAE and Sham groups. A total of 581 DEPs were identified, including 429 upregulated and 152 downregulated proteins. **(D)** Volcano plot showing DEPs between the SAE + NaHCO₃ and SAE groups. A total of 271 DEPs were identified, including 78 upregulated and 193 downregulated proteins. DEPs were identified using Student’s t-test with a nominal *p* value < 0.05 and a fold-change threshold of >1.20 or <0.83. Red and blue indicate upregulated and downregulated proteins, respectively.

Together, these findings demonstrate substantial transcriptomic and proteomic remodeling in the hippocampus during SAE and further molecular alterations following NaHCO₃ treatment.

### 3.3 Integrated transcriptomic and proteomic analyses reveal cross-omics molecular changes associated with SAE and NaHCO₃ treatment

To further examine molecular changes shared across the transcriptomic and proteomic datasets, pairwise cross-omics integration was performed for the SAE versus Sham and SAE + NaHCO₃ versus SAE comparisons. In the SAE versus Sham comparison, overlap analysis identified 57 molecules showing concordant differential changes at both the transcript and protein levels, including 49 concordantly upregulated and 8 concordantly downregulated molecules (Figure 4A; Supplementary Table S7). In the SAE + NaHCO₃ versus SAE comparison, four molecules showed concordant downregulation at both omics levels (Figure 4B; Supplementary Table S8).

**Figure 4:**
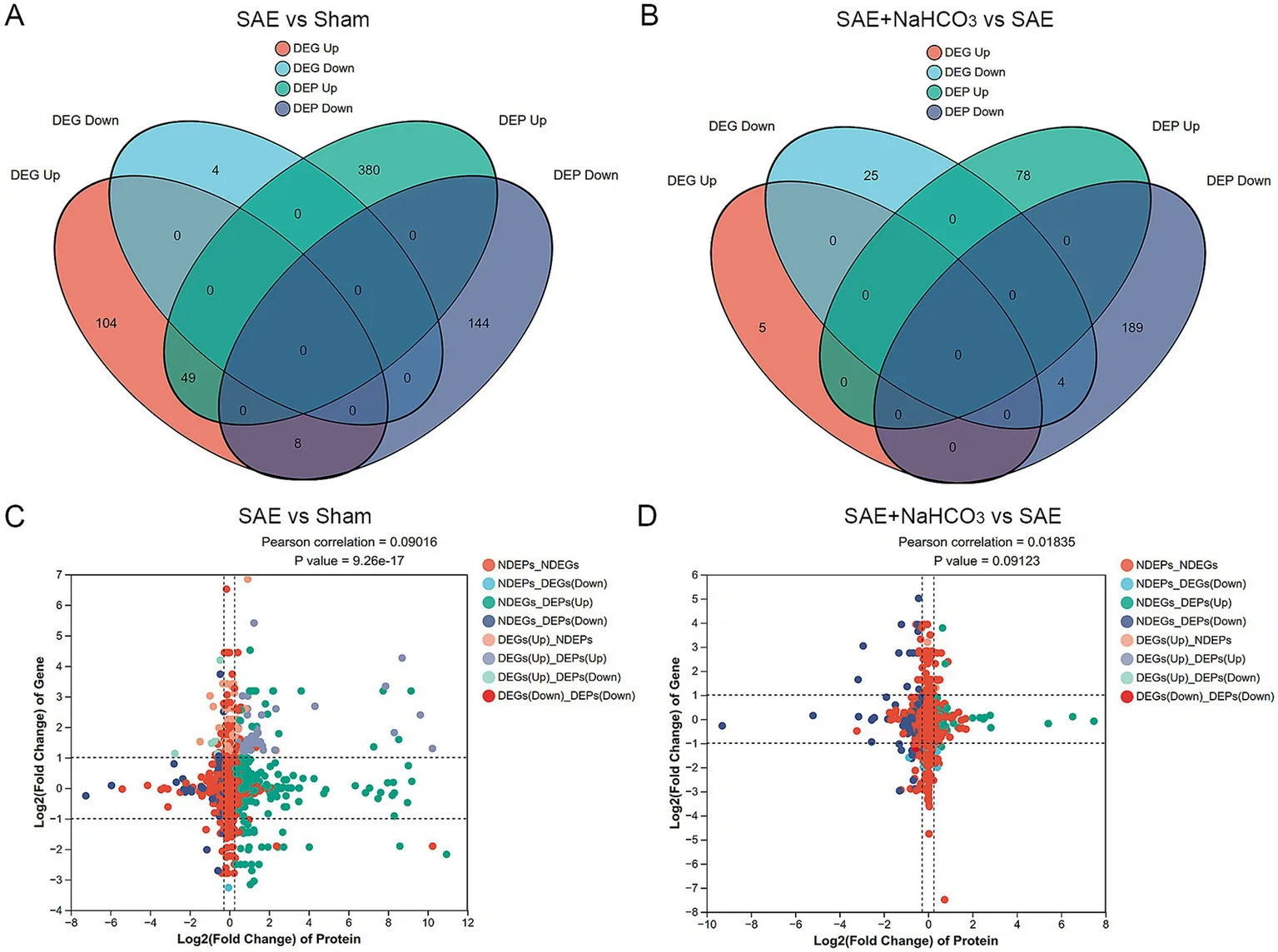
Pairwise cross-omics integration reveals shared transcriptomic and proteomic alterations associated with SAE and NaHCO₃ treatment. **(A)** Cross-omics overlap between differentially expressed genes (DEGs) and differentially expressed proteins (DEPs) in the SAE versus Sham comparison. A total of 57 overlapping RNA–protein entries were identified, including 49 showing concordant upregulation and 8 showing concordant downregulation at both the transcriptomic and proteomic levels. **(B)** Cross-omics overlap in the SAE + NaHCO₃ versus SAE comparison, identifying four overlapping RNA–protein entries, all of which showed concordant downregulation at both omics levels. **(C)** Correlation between transcriptomic and proteomic fold changes in the SAE versus Sham comparison (Pearson *r* = 0.090, *p* = 9.26 × 10^−17^). **(D)** Correlation between transcriptomic and proteomic fold changes in the SAE + NaHCO₃ versus SAE comparison (Pearson *r* = 0.018, *p* = 0.091). Detailed overlapping entries are provided in Supplementary Tables S7, S8.

We next examined the overall relationship between transcriptomic and proteomic fold changes. A weak but statistically significant positive correlation was observed for the SAE versus Sham comparison (Pearson *r* = 0.090, *p* < 0.001; Figure 4C), whereas no significant overall correlation was observed for the SAE + NaHCO₃ versus SAE comparison (Pearson *r* = 0.018, *p* = 0.091; Figure 4D). Consistent with these findings, global transcript–protein correlation and matched expression patterns are provided in Supplementary Figure S2.

These results indicate that a subset of molecular alterations is concordantly detected at both transcript and protein levels, although the overall correspondence between the two omics layers is limited.

### 3.4 Functional enrichment analysis reveals immune- and inflammation-related molecular alterations in SAE

To characterize the biological processes underlying SAE-associated molecular changes, GO and KEGG enrichment analyses were performed. GO enrichment analysis of the SAE versus Sham comparison revealed prominent alterations in biological processes associated with immune regulation, inflammatory responses, and related cellular functions at the transcriptomic and/or proteomic levels (Figure 5A; Supplementary Table S9). The SAE + NaHCO₃ versus SAE comparison also showed changes in multiple immune- and inflammation-related biological processes (Figure 5B; Supplementary Table S10).

**Figure 5:**
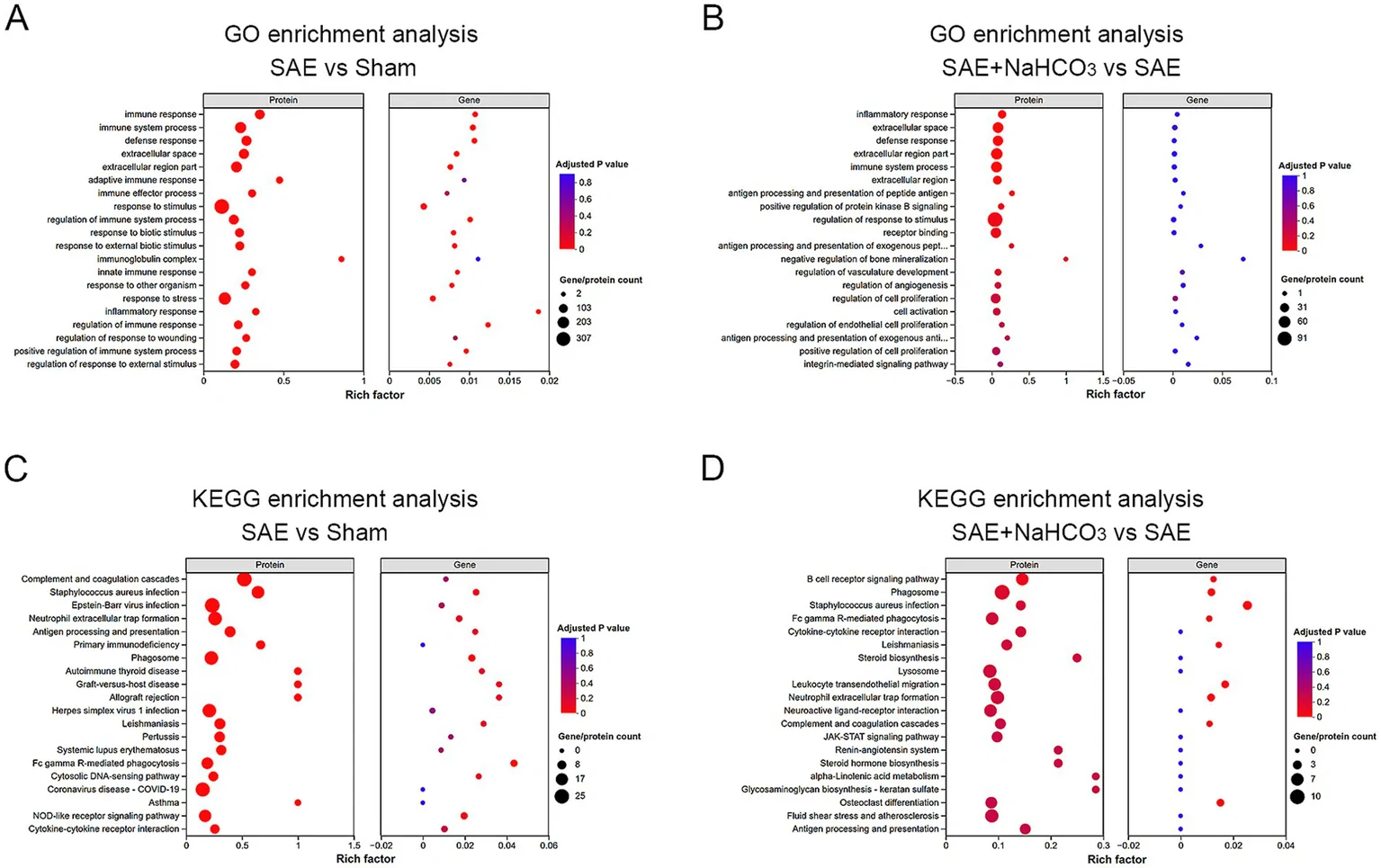
Functional enrichment analysis of transcriptomic and proteomic alterations associated with SAE and NaHCO₃ treatment. **(A)** Gene Ontology (GO) enrichment analysis of molecular alterations identified in the SAE versus Sham comparison at the transcriptomic and proteomic levels. **(B)** GO enrichment analysis of molecular alterations identified in the SAE + NaHCO₃ versus SAE comparison. **(C)** Kyoto Encyclopedia of Genes and Genomes (KEGG) pathway enrichment analysis of the SAE versus Sham comparison. **(D)** KEGG pathway enrichment analysis of the SAE + NaHCO₃ versus SAE comparison. Transcriptomic and proteomic enrichment results are displayed in parallel to facilitate comparison of functional alterations across the two omics layers. Detailed enrichment results are provided in Supplementary Tables S9–S12.

KEGG pathway analysis further identified enrichment of several immune- and inflammation-associated pathways in SAE relative to Sham (Figure 5C; Supplementary Table S11). Following NaHCO₃ treatment, changes were also observed in several immune-related pathways relative to untreated SAE mice (Figure 5D; Supplementary Table S12). Complementary GSVA and pathway-level transcriptome–proteome analyses further demonstrated coordinated functional alterations across the two omics layers (Supplementary Figure S3).

Together, these analyses suggest that SAE-associated hippocampal molecular remodeling prominently involves immune- and inflammation-related biological processes and that NaHCO₃ treatment is accompanied by alterations in several of these functional pathways.

### 3.5 Three-group cross-omics trajectory analysis prioritizes C3 as a candidate molecule associated with SAE and NaHCO₃ treatment

To identify candidate molecules showing coordinated changes across the three experimental groups, we next integrated the transcriptomic and proteomic datasets using a three-group trajectory-based analysis. This analysis identified 18 common candidates exhibiting corresponding expression trajectories across the transcriptomic and proteomic datasets (Figures 6A,B; Supplementary Table S13).

**Figure 6:**
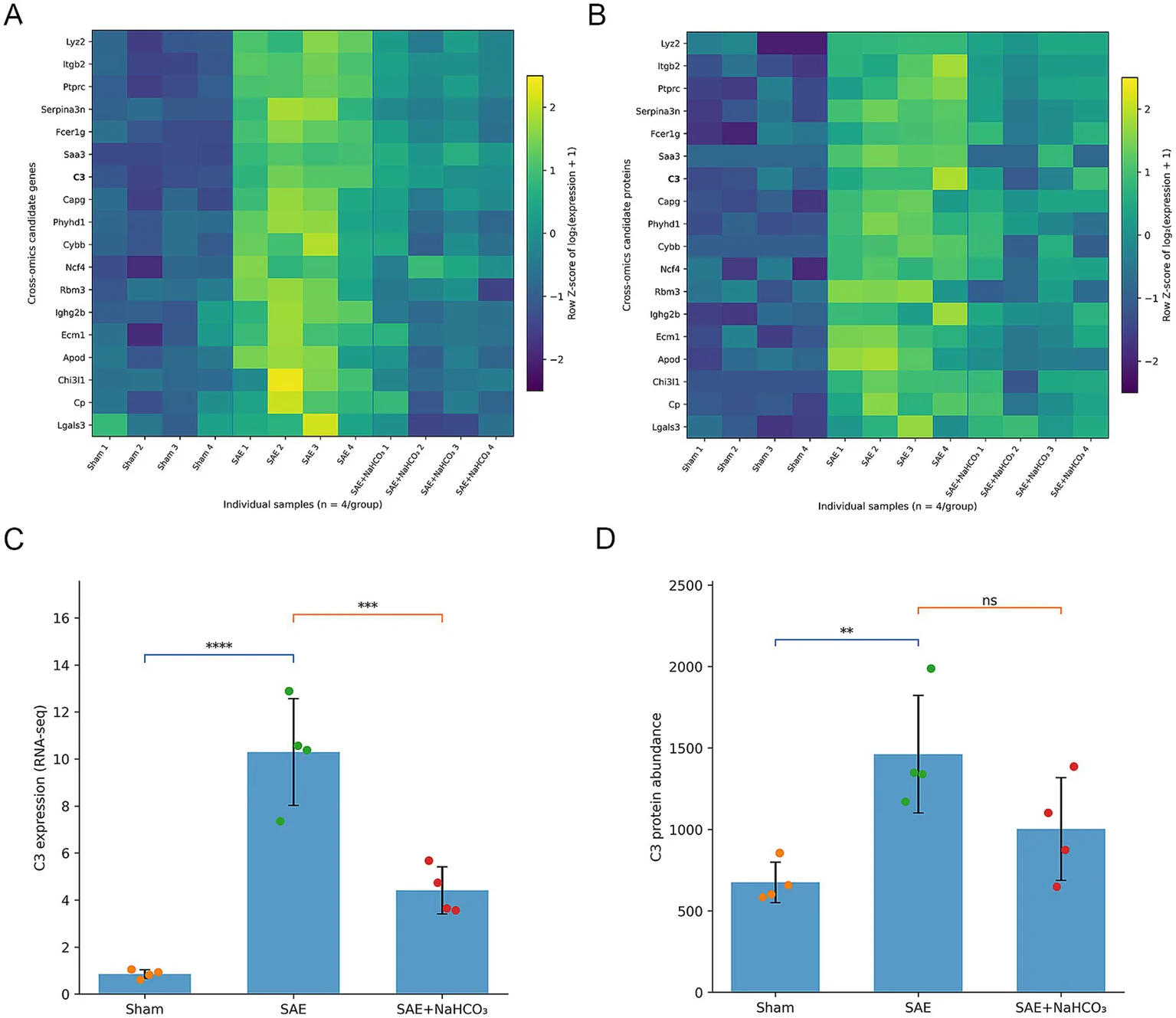
Three-group cross-omics trajectory analysis identifies candidate molecules associated with SAE and NaHCO₃ treatment and prioritizes C3 for subsequent analysis. **(A,B)** Heatmaps showing the expression patterns of 18 common candidate molecules identified at the transcriptomic **(A)** and proteomic **(B)** levels across the Sham, SAE, and SAE + NaHCO₃ groups. Candidates were retained when they showed an FDR < 0.05 at both the transcriptomic and proteomic levels and a concordant three-group expression trajectory characterized by an SAE-associated increase followed by a decrease after NaHCO₃ treatment. **(C)** RNA-seq-derived C3 expression across the three experimental groups. **(D)** Quantitative proteomics-derived C3 abundance across the three groups. C3 showed a significant SAE-associated increase at both omics levels. At the transcriptomic level, C3 was significantly decreased following NaHCO₃ treatment, whereas at the proteomic level, the decrease following treatment did not reach statistical significance in the pairwise *post-hoc* comparison. Data in C and D are presented as mean ± SD. Statistical significance was assessed using one-way ANOVA followed by Tukey’s multiple-comparison test for the displayed C3 group comparisons. *p < 0.05; ***p < 0.001; ****p < 0.0001; ns, not significant. The complete list and statistical information for the 18 candidates are provided in Supplementary Table S13.

Among these candidates, C3 was prioritized for further investigation based on its consistent cross-omics expression pattern and its established relevance to complement-mediated neuroimmune responses. At the transcript level, C3 expression was significantly increased in SAE mice compared with Sham controls and significantly decreased following NaHCO₃ treatment (Figure 6C). At the protein level, C3 abundance was significantly increased in SAE mice compared with Sham controls. Although C3 protein abundance showed a downward trend following NaHCO₃ treatment, the pairwise *post-hoc* comparison between SAE and SAE + NaHCO₃ groups did not reach statistical significance (Figure 6D).

These findings identify C3 as a cross-omics candidate associated with SAE-related hippocampal molecular alterations and support its prioritization for subsequent targeted analysis.

### 3.6 Validation of hippocampal C3 expression changes identified by multi-omics analysis

To validate the multi-omics findings, hippocampal C3 expression was examined using qRT-PCR and Western blot analysis. Consistent with RNA-sequencing results, qRT-PCR revealed significantly increased C3 mRNA expression in SAE mice compared with Sham controls, while NaHCO₃ treatment markedly reduced C3 transcript levels (Figure 7A). Western blot analysis demonstrated a similar pattern at the protein level. Hippocampal C3 protein abundance was elevated following SAE induction and was significantly decreased after NaHCO₃ administration (Figure 7B).

**Figure 7:**
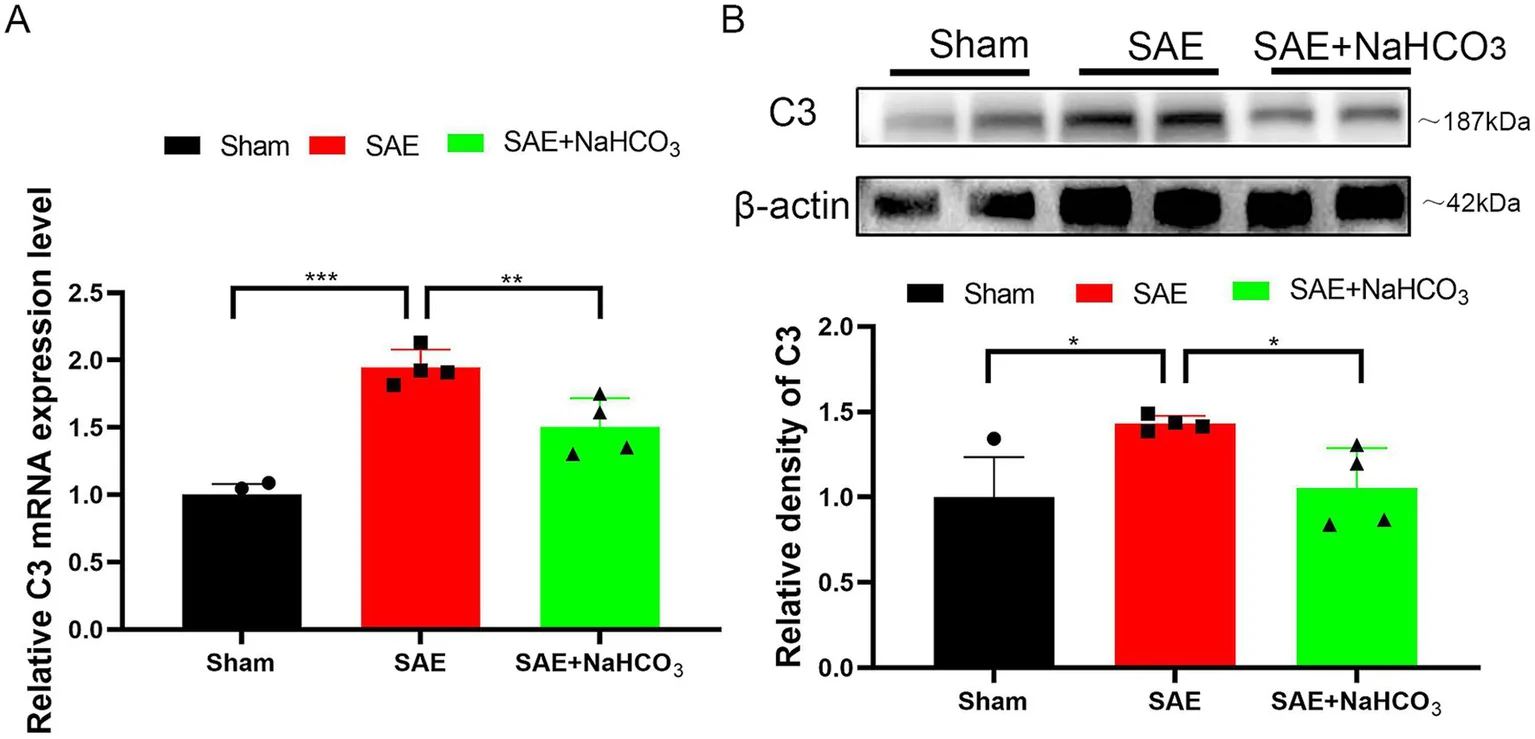
Targeted assessment of hippocampal C3 expression following SAE induction and NaHCO₃ treatment. **(A)** Quantitative real-time PCR (qRT-PCR) analysis of relative C3 mRNA expression in hippocampal tissues from the Sham, SAE, and SAE + NaHCO₃ groups (*n* = 4 biological replicates/group). **(B)** Representative Western blot and densitometric analysis of the C3-immunoreactive band at approximately 187 kDa in hippocampal tissues (*n* = 4 biological replicates/group). C3 protein abundance was normalized to β-actin. Data are presented as mean ± SD. Statistical significance was assessed using one-way ANOVA followed by Tukey’s multiple-comparison test. *p < 0.05; **p < 0.01; ***p < 0.001; ****p < 0.0001, as indicated in the corresponding panels.

These findings confirm that hippocampal C3 expression is enhanced during SAE and is responsive to NaHCO₃ treatment.

### 3.7 Hippocampal C3 shows increased spatial association with GFAP-positive astrocytic structures during SAE

Because C3 has been implicated in inflammatory astrocytic responses, we next examined the spatial relationship between C3 and GFAP-positive astrocytic structures in the hippocampus. Immunofluorescence staining revealed enhanced C3 immunoreactivity in SAE mice, accompanied by increased spatial overlap between C3 and GFAP signals (Figures 8A–C). Quantitative colocalization analysis showed an increased Pearson’s correlation coefficient between C3 and GFAP signals following SAE induction, indicating enhanced spatial association between the two signals; this increase was attenuated following NaHCO₃ treatment (Figure 8D). Manders’ coefficient analysis further showed an increased proportion of C3 signal overlapping with GFAP-positive structures (M1) in SAE mice, which was reduced following NaHCO₃ treatment (Figure 8E). In contrast, the proportion of GFAP signal overlapping with C3-positive structures (M2) did not differ significantly between the SAE and SAE + NaHCO₃ groups (Figure 8F).

**Figure 8:**
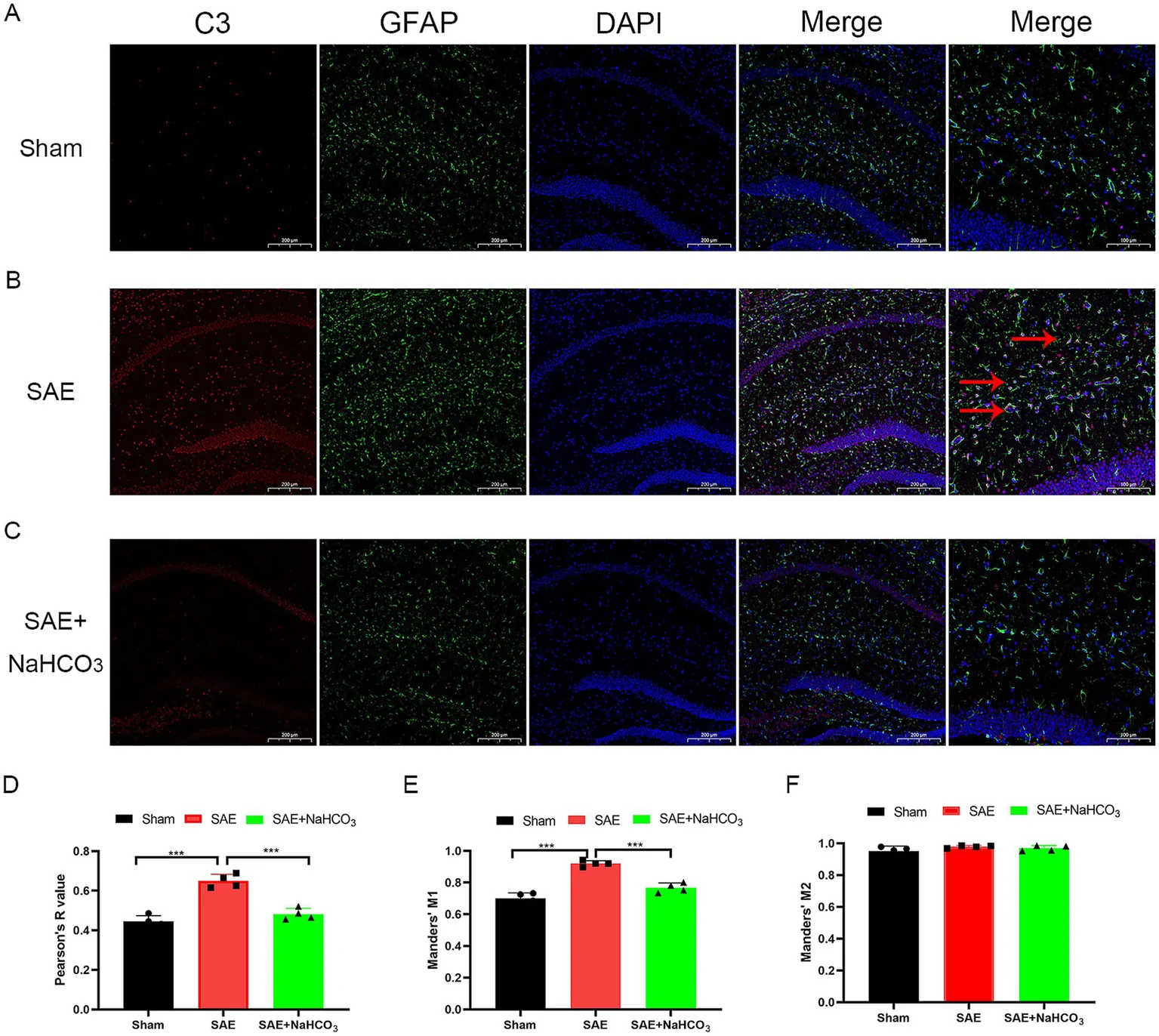
NaHCO₃ attenuates the increased spatial association between C3 and GFAP-positive astrocytic structures in the hippocampus of SAE mice. **(A–C)** Representative immunofluorescence images of hippocampal sections stained for GFAP (astrocytes, green), C3 (red), and DAPI (blue). Scale bars are shown in the images. **(D)** Quantitative analysis of the Pearson’s correlation coefficient for GFAP and C3 fluorescence signals. **(E)** Quantitative analysis of Manders’ overlap coefficient M1 (fraction of C3 signal overlapping GFAP). **(F)** Quantitative analysis of Manders’ overlap coefficient M2 (fraction of GFAP signal overlapping C3). Immunofluorescence analysis was performed using four animals per group (*n* = 4 biological replicates/group), with three microscopic fields analyzed per animal. Measurements from the three fields of each animal were averaged to generate a single animal-level value for statistical analysis. Data are presented as mean ± SD. Statistical analyses were performed using one-way ANOVA followed by Tukey’s multiple-comparison test. ***p < 0.001. Scale bars = 100 μm.

Together, these findings demonstrate increased spatial association between hippocampal C3 and GFAP-positive astrocytic structures during SAE, which was attenuated following NaHCO₃ treatment. These colocalization data indicate spatial association but do not establish the cellular source or causal role of C3.

## 4 Discussion

Sepsis-associated encephalopathy (SAE) is a complex neurological complication of sepsis characterized by cognitive impairment and persistent brain dysfunction (Shankar-Hari et al., 2026a; Shankar-Hari et al., 2026b). In this study, we demonstrated that sodium bicarbonate (NaHCO₃) treatment improved survival and cognitive deficits in an LPS-induced SAE mouse model. Separate transcriptomic and proteomic profiling, followed by cross-omics integration, revealed extensive hippocampal molecular remodeling associated with SAE and identified 18 common candidate molecules showing significant alterations at both omics levels and a concordant SAE-associated increase followed by a decrease after NaHCO₃ treatment. Among these candidates, complement component 3 (C3) was prioritized for subsequent targeted analysis based on its cross-omics expression pattern and biological relevance to neuroimmune responses. Further targeted assessment confirmed increased hippocampal C3 expression at both transcript and protein levels, accompanied by enhanced spatial association between C3 and GFAP signals. These findings suggest that C3-associated alterations involving GFAP-positive astrocytic structures may represent a potential component of hippocampal neuroimmune remodeling during SAE and show that these alterations are responsive to NaHCO₃ treatment.

Growing evidence indicates that SAE is not solely caused by circulating inflammatory mediators but results from complex interactions between systemic inflammation and central nervous system responses (Wang X. et al., 2026). During sepsis, disruption of blood–brain barrier integrity, immune activation, glial reactivity, and altered neuronal homeostasis collectively contribute to cognitive dysfunction (Jia et al., 2026; Peng et al., 2026). The hippocampus is particularly vulnerable to inflammatory insults because of its critical role in learning and memory (Chen et al., 2025; Leemburg et al., 2025). Integrated multi-omics approaches have provided complementary insights into molecular alterations associated with neuroinflammatory disorders (Hu et al., 2026). In the present study, our multi-omics analyses demonstrated broad molecular alterations in the hippocampus following SAE induction, with enrichment of immune-related and inflammatory pathways. These findings support the concept that hippocampal neuroimmune dysregulation represents an important molecular feature of SAE. Importantly, NaHCO₃ treatment was accompanied by partial reversal of several SAE-associated molecular alterations, suggesting that modulation of neuroimmune homeostasis may be associated with its protective effects.

The complement system has recently been recognized as an important regulator of neuroimmune interactions beyond its classical function in innate immunity (Mallah et al., 2025; Chacon et al., 2026). Among complement components, C3 serves as a central convergence molecule and has been implicated in inflammatory responses, synaptic remodeling, and glial activation in various neurological disorders (Gao et al., 2025; Lu et al., 2026). In particular, increased C3 expression has been associated with a subset of reactive astrocytic states characterized by altered inflammatory profiles (He et al., 2025; Peng et al., 2025). In the present study, three-group cross-omics analysis identified C3 among 18 candidate molecules meeting the FDR < 0.05 criterion at both the transcriptomic and proteomic levels and showing a concordant SAE-associated increase followed by a decrease after NaHCO₃ treatment. C3 was subsequently prioritized for targeted analysis because of its cross-omics expression pattern and established relevance to neuroimmune processes. Targeted qRT-PCR and Western blot analyses further supported increased hippocampal C3 expression following SAE induction and lower C3 expression after NaHCO₃ treatment. Furthermore, immunofluorescence analysis demonstrated increased spatial association between C3 and GFAP-positive astrocytic structures in SAE mice, which was attenuated after NaHCO₃ treatment. Together, these findings suggest that C3-associated alterations involving GFAP-positive astrocytic structures may represent an important neuroimmune feature of SAE. However, the present colocalization data do not establish astrocytes as the cellular source of C3 or demonstrate a causal relationship between C3–GFAP spatial association and neurological dysfunction, and future studies using astrocyte-specific C3 manipulation are required to determine whether this pathway directly contributes to neuronal dysfunction and cognitive impairment.

NaHCO₃ is traditionally used as an alkalinizing agent for correcting metabolic disturbances; however, accumulating evidence suggests that bicarbonate signaling may influence biological processes beyond acid–base regulation (Liu et al., 2025; Marsh et al., 2026). Changes in extracellular pH, cellular metabolism, and inflammatory signaling can affect immune cell function and tissue homeostasis under pathological conditions (Alvarez et al., 2023; Neale et al., 2024; Ma et al., 2025). In sepsis, metabolic imbalance and excessive inflammatory activation are closely interconnected and contribute to neuronal dysfunction. Our findings suggest that NaHCO₃ treatment is associated with partial normalization of several inflammation- and immune-related hippocampal molecular alterations. The concurrent changes in hippocampal C3 expression and attenuation of C3/GFAP spatial association following treatment further indicate that complement-associated molecular alterations involving astrocytic structures are responsive to NaHCO₃ intervention. However, blood pH, circulating bicarbonate, lactate, and other systemic metabolic parameters were not measured. Consequently, our data cannot distinguish whether these observations resulted primarily from correction of systemic acid–base disturbances, secondary metabolic effects, direct immunomodulatory actions, or a combination of these processes. Therefore, the present findings should be interpreted as identifying molecular changes associated with NaHCO₃ intervention rather than establishing a direct neuroimmune mechanism of NaHCO₃ action.

This study provides several important insights into molecular alterations associated with SAE. Unlike previous studies mainly focusing on systemic inflammatory mediators or microglial activation, our work highlights complement-associated alterations involving GFAP-positive astrocytic structures in the hippocampus during sepsis. By integrating transcriptomic and proteomic analyses with targeted molecular and cellular assessments, we identified C3 as one of 18 cross-omics candidate molecules and subsequently prioritized it for further investigation based on its expression pattern and neuroimmune relevance. These findings expand the current understanding of SAE pathogenesis and suggest that astrocyte-associated complement responses may represent an important component of sepsis-induced brain dysfunction. Importantly, although C3 was identified as a candidate neuroimmune-associated molecule, our study does not define C3 as an independent therapeutic target. Rather, the findings provide a framework for future investigation of complement–astrocyte interactions in SAE.

Several limitations should be considered. First, although integrated multi-omics analysis and subsequent targeted molecular assessment identified C3 as one of the candidate molecules associated with SAE and NaHCO₃ responsiveness, the present study did not include C3 loss- or gain-of-function experiments. Therefore, a causal role for C3 in SAE-associated brain dysfunction or in the beneficial effects observed following NaHCO₃ treatment cannot be established. Future studies employing C3 knockout, pharmacological inhibition, overexpression, and particularly cell-type-specific C3 manipulation will be necessary to determine its functional contribution to SAE pathophysiology. In addition, although C3 showed increased spatial association with GFAP-positive structures, the present colocalization analysis cannot establish astrocytes as the cellular source of C3; cell-type-specific approaches will be required to resolve its cellular origin. Second, systemic acid–base and metabolic parameters, including blood pH, circulating bicarbonate, and lactate levels, were not measured in the present study. Therefore, we cannot determine whether the effects associated with NaHCO₃ administration resulted from correction of systemic acid–base disturbances, secondary metabolic changes, direct immunomodulatory effects, or their combination. Future studies incorporating serial blood gas and metabolic measurements together with neuroinflammatory assessments will be necessary to clarify the physiological basis of the observed effects. Finally, the LPS-induced SAE model reproduces important inflammatory features of sepsis but does not fully represent the complexity of human disease. Validation in clinically relevant sepsis models and human samples will be required to determine the translational significance of C3-associated neuroimmune alterations.

## 5 Conclusion

NaHCO₃ treatment alleviated survival impairment and cognitive dysfunction and was associated with altered hippocampal neuroimmune profiles in an LPS-induced SAE mouse model. Integrated three-group transcriptomic and proteomic analysis identified 18 cross-omics candidate molecules showing significant alterations at both omics levels and a concordant SAE-associated increase followed by a decrease after NaHCO₃ treatment, among which C3 was prioritized for further investigation. Targeted molecular analyses supported increased hippocampal C3 expression during SAE and its responsiveness to NaHCO₃ treatment. The increased spatial association between C3 and GFAP-positive astrocytic structures further suggests that complement-associated alterations accompany astrocytic responses during SAE. However, the causal contribution of C3 and the physiological mechanisms underlying the effects associated with NaHCO₃ remain to be determined.

## Data Availability

The datasets presented in this study can be found in online repositories. The names of the repository/repositories and accession number(s) can be found in the article/Supplementary material.

## Supplementary material

The Supplementary material for this article can be found online at:

https://www.frontiersin.org/articles/10.3389/fnins.2026.1954272/full#supplementary-material

## References

[ref1] Alvarez M. R. Alarcon J. M. Roman C. A. Lazaro D. Bobrowski-Khoury N. Baena-Caldas G. P. . (2023). Can a basic solution activate the inflammatory reflex? A review of potential mechanisms, opportunities, and challenges. Pharmacol. Res. 187:106525. doi: 10.1016/j.phrs.2022.106525 36441036

[ref2] Bastola T. Perkins G. A. Huu V.a. N. Ju S. Kim K. Y. Shen Z. . (2024). Administration of bicarbonate protects mitochondria, rescues retinal ganglion cells, and ameliorates visual dysfunction caused by oxidative stress. Antioxidants 13:743. doi: 10.3390/antiox13060743, 38929182 PMC11200884

[ref3] Chacon A. Khelo R. R. Saada L. J. M. Grossberg J. A. Reisner A. Alawieh A. M. . (2026). Complement in traumatic brain injury: linking acute injury to chronic neurodegeneration. Eur. J. Immunol. 56:e70230. doi: 10.1002/eji.70230, 42319149 PMC13473809

[ref4] Chen T. Hu J. Liao Y. Xie S. Zhang L. (2025). The brain washing system in sepsis-associated encephalopathy. J. Neuroinflammation 22:277. doi: 10.1186/s12974-025-03602-4, 41287063 PMC12642026

[ref5] Cheng J. Zheng Y. Cheng F. Wang C. Han J. Zhang H. . (2026). Different roles of astrocytes in the blood-brain barrier during the acute and recovery phases of stroke. Neural Regen. Res. 21, 1359–1372. doi: 10.4103/nrr.Nrr-d-24-01417, 40537147 PMC12407517

[ref6] Escartin C. Galea E. Lakatos A. O'callaghan J. P. Petzold G. C. Serrano-Pozo A. . (2021). Reactive astrocyte nomenclature, definitions, and future directions. Nat. Neurosci. 24, 312–325. doi: 10.1038/s41593-020-00783-4 PMC8007081 33589835

[ref7] Fisher T. M. Liddelow S. A. (2024). Emerging roles of astrocytes as immune effectors in the central nervous system. Trends Immunol. 45, 824–836. doi: 10.1016/j.it.2024.08.008, 39332912

[ref8] Gao J. Lin C. Deng L. Wang H. Wang X. (2025). Nanomolar TLR4 antagonist CIAC101 derived from (+)-naltrexone blocks microglial activation and methamphetamine addiction. J. Med. Chem. 68, 25469–25484. doi: 10.1021/acs.jmedchem.5c02596, 41265859

[ref9] Gofton T. E. Young G. B. (2012). Sepsis-associated encephalopathy. Nat. Rev. Neurol. 8, 557–566. doi: 10.1038/nrneurol.2012.183, 22986430

[ref10] He R. Wu B. Han L. Li M. Guo W. Fu J. . (2025). Activation of spinal astrocyte α2A Adrenoceptors protects against Sepsis-induced heart injury through inhibition of GABAergic neuronal necroptosis. Adv. Sci. 12:e04406. doi: 10.1002/advs.202504406, 40692211 PMC12533331

[ref11] Hong S. Beja-Glasser V. F. Nfonoyim B. M. Frouin A. Li S. Ramakrishnan S. . (2016). Complement and microglia mediate early synapse loss in Alzheimer mouse models. Science 352, 712–716. doi: 10.1126/science.aad8373, 27033548 PMC5094372

[ref12] Hu L. Cheng C. Pu J. C. Huang C. L. Li T. Wen F. . (2026). Multi-omics profiling of cerebrospinal fluid in autoimmune encephalitis: insights into pathogenesis and therapeutic targets. Brain Behav. Immun. 137:106822. doi: 10.1016/j.bbi.2026.106822, 42162799

[ref13] Jia M. Shao H. Ma S.-Q. Liu W.-X. Cai M. Zhu T. . (2026). Microglial galectin-3 disrupts parvalbumin interneurons and hippocampal synchrony, driving cognitive deficits. J. Neuroinflammation. 23:276. doi: 10.1186/s12974-026-03889-x PMC13483578 42249483

[ref14] Leemburg S. Kala A. Nataraj A. Karkusova P. Baindur S. Suresh A. . (2025). LPS-induced systemic inflammation disrupts brain activity in a region- and vigilance-state specific manner. Brain Behav. Immun. 128, 713–724. doi: 10.1016/j.bbi.2025.05.002 40349731

[ref15] Liddelow S. A. Guttenplan K. A. Clarke L. E. Bennett F. C. Bohlen C. J. Schirmer L. . (2017). Neurotoxic reactive astrocytes are induced by activated microglia. Nature 541, 481–487. doi: 10.1038/nature21029, 28099414 PMC5404890

[ref16] Liu S. Gao Y. Luo J. Chen Y. Cao H. Guo Z. . (2025). Dietary baking soda (NaHCO3) therapy recovered urolithiasis-induced kidney injury in mice by inhibition of oxidative stress, pyroptosis, and inflammation through gut-kidney axis. Ren. Fail. 47:2521456. doi: 10.1080/0886022x.2025.2521456, 40583356 PMC12210412

[ref17] Lu X. Jiang Y. Lin X. Zhang Y. Liu Q. Chen S. (2026). Porcine plasma-derived extracellular vesicles orchestrate multi-target neuroimmune reconfiguration to alleviate Alzheimer's disease pathology in a 5×FAD mouse model. J. Neuroinflammation 23:234. doi: 10.1186/s12974-026-03866-4, 42120990 PMC13343742

[ref18] Ma L. Wang X. Xu H. (2025). Mitochondrial DNA damage in HIV infection: a mechanistic driver of immunometabolic dysfunction and chronic inflammation. Front. Immunol. 16:1722463. doi: 10.3389/fimmu.2025.1722463, 41601636 PMC12832992

[ref19] Mallah K. Krieg C. Hatchell D. Hamouda N. Roof T. Walterhouse S. . (2025). Targeted complement inhibition ameliorates the pathological and cognitive outcomes in repetitive mild closed head injury. Signal Transduct. Target. Ther. 10:378. doi: 10.1038/s41392-025-02466-7, 41261092 PMC12630812

[ref20] Marsh K. M. Rabinovich E. P. Rastogi R. Zhang A. Sawyer R. G. Yang Z. (2026). Topical bicarbonate therapy to the spleen improves survival in rats with severe intra-abdominal sepsis. Surg. Infect. 27, 145–152. doi: 10.1177/10962964251394205, 41187980

[ref21] Mazeraud A. Righy C. Bouchereau E. Benghanem S. Bozza F. A. Sharshar T. (2020). Septic-associated encephalopathy: a comprehensive review. Neurotherapeutics 17, 392–403. doi: 10.1007/s13311-020-00862-1, 32378026 PMC7283452

[ref22] Mohebbi N. Ritter A. Wiegand A. Graf N. Dahdal S. Sidler D. . (2023). Sodium bicarbonate for kidney transplant recipients with metabolic acidosis in Switzerland: a multicentre, randomised, single-blind, placebo-controlled, phase 3 trial. Lancet 401, 557–567. doi: 10.1016/s0140-6736(22)02606-x, 36708734

[ref23] Mou W. Ma L. Zhu A. Cui H. Huang Y. (2022). Astrocyte-microglia interaction through C3/C3aR pathway modulates neuropathic pain in rats model of chronic constriction injury. Mol. Pain 18:17448069221140532. doi: 10.1177/17448069221140532, 36341694 PMC9669679

[ref24] Neale I. Reddy C. Tan Z. Y. Li B. Nag P. P. Park J. . (2024). Small-molecule probe for IBD risk variant GPR65 I231L alters cytokine signaling networks through positive allosteric modulation. Sci. Adv. 10:eadn2339. doi: 10.1126/sciadv.adn2339, 39028811 PMC11259170

[ref25] Ogunleye F. A. Zhou Y. Yan L. Abhulimen P. O. Xu Y. Lai M. K. P. . (2026). Metabolic and inflammatory roles of glial cells in neurodegenerative and cerebrovascular diseases. Ageing Res. Rev. 118:103120. doi: 10.1016/j.arr.2026.103120, 41932503

[ref26] O'neill L. A. Kishton R. J. Rathmell J. (2016). A guide to immunometabolism for immunologists. Nat. Rev. Immunol. 16, 553–565. doi: 10.1038/nri.2016.70 PMC5001910 27396447

[ref27] Peng M. Chen W. Gao Q. Zhu X. Yu L. Zhang L. . (2026). Enhanced autophagy drives endothelial tight junction loss, BBB disruption, and behavioral deficits during inflammation. Autophagy 22, 1852–1869. doi: 10.1080/15548627.2026.2662440, 42003242 PMC13449731

[ref28] Peng X. Ju J. Li Z. Liu J. Jia X. Wang J. . (2025). C3/C3aR bridges spinal astrocyte-microglia crosstalk and accelerates Neuroinflammation in morphine-tolerant rats. CNS Neurosci. Ther. 31:e70216. doi: 10.1111/cns.70216, 39801259 PMC11725764

[ref29] Prescott H. C. Angus D. C. (2018). Enhancing recovery from Sepsis: a review. JAMA 319, 62–75. doi: 10.1001/jama.2017.17687, 29297082 PMC5839473

[ref30] Prescott H. C. Antonelli M. Alhazzani W. Møller M. H. Alshamsi F. Azevedo L. C. P. . (2026). Surviving Sepsis campaign: international guidelines for Management of Sepsis and Septic Shock 2026. Crit. Care Med. 54, 725–812. doi: 10.1097/ccm.0000000000007075, 41869847

[ref31] Ricklin D. Hajishengallis G. Yang K. Lambris J. D. (2010). Complement: a key system for immune surveillance and homeostasis. Nat. Immunol. 11, 785–797. doi: 10.1038/ni.1923, 20720586 PMC2924908

[ref32] Semmler A. Hermann S. Mormann F. Weberpals M. Paxian S. A. Okulla T. . (2008). Sepsis causes neuroinflammation and concomitant decrease of cerebral metabolism. J. Neuroinflammation 5:38. doi: 10.1186/1742-2094-5-38, 18793399 PMC2553764

[ref33] Shankar-Hari M. Benghanem S. Bourhy L. Cunningham C. Ehler J. Girard T. D. . (2026a). Current knowledge and challenges of sepsis-associated encephalopathy. Intensive Care Med. 52, 1679–1696 doi: 10.1007/s00134-026-08489-0 42301312

[ref34] Shankar-Hari M. Benghanem S. Bourhy L. Cunningham C. Ehler J. Girard T. D. . (2026b). Publisher correction: current knowledge and challenges of sepsis-associated encephalopathy. Intensive Care Med. 52:1826 doi: 10.1007/s00134-026-08547-7 42390594

[ref35] Sofroniew M. V. (2015). Astrocyte barriers to neurotoxic inflammation. Nat. Rev. Neurosci. 16, 249–263. doi: 10.1038/nrn3898, 25891508 PMC5253239

[ref36] Tian X. Zhang L. Qian X. Peng Y. Chen F. Bengtson S. . (2025). Gut complement system: a new frontier in microbiota-host communication and intestinal homeostasis. J. Clin. Invest. 135:e188349. doi: 10.1172/jci188349, 41031883 PMC12483559

[ref37] Wang D. Jin K. Ying C. Li B. Zhang G. Li J. . (2026). Intratumoral bicarbonate functions as an adjuvant to potentiate PD-1 blockade in hepatocellular carcinoma. Oncogene 45, 2683–2698. doi: 10.1038/s41388-026-03836-3, 42243329

[ref38] Wang X. Liu Y. Li M. Xu H. Ju Y. Cui L. . (2026). Ultrasound-gated nanobubbles for sequential pyroptosis blockade and mechanotransductive neurorepair in sepsis-associated encephalopathy. Biomaterials 336:124475. doi: 10.1016/j.biomaterials.2026.124475, 42503286

[ref39] Wang L. Zhu J. Ding Z. Xie Z. Liu X. Zhang F. . (2026). Immune predictors of radiotherapy outcomes in cervical cancer. Adv. Sci. 13:e09784. doi: 10.1002/advs.202509784, 41560673 PMC13042687

