# Supplementary material for: Integrated transcriptomic and proteomic profiling reveals C3-associated hippocampal neuroimmune remodeling in sodium bicarbonate-treated sepsis-associated encephalopathy
Source: Front Neurosci. 2026 Oct 7;20:1954272. doi: 10.3389/fnins.2026.1954272 (PMC13684795; doi:10.3389/fnins.2026.1954272)
Supplement: Supplementary file 1 [file Data_Sheet_12.PDF]

Raw image for Figure 7B (β-Actin internal control)

~42(kDa)

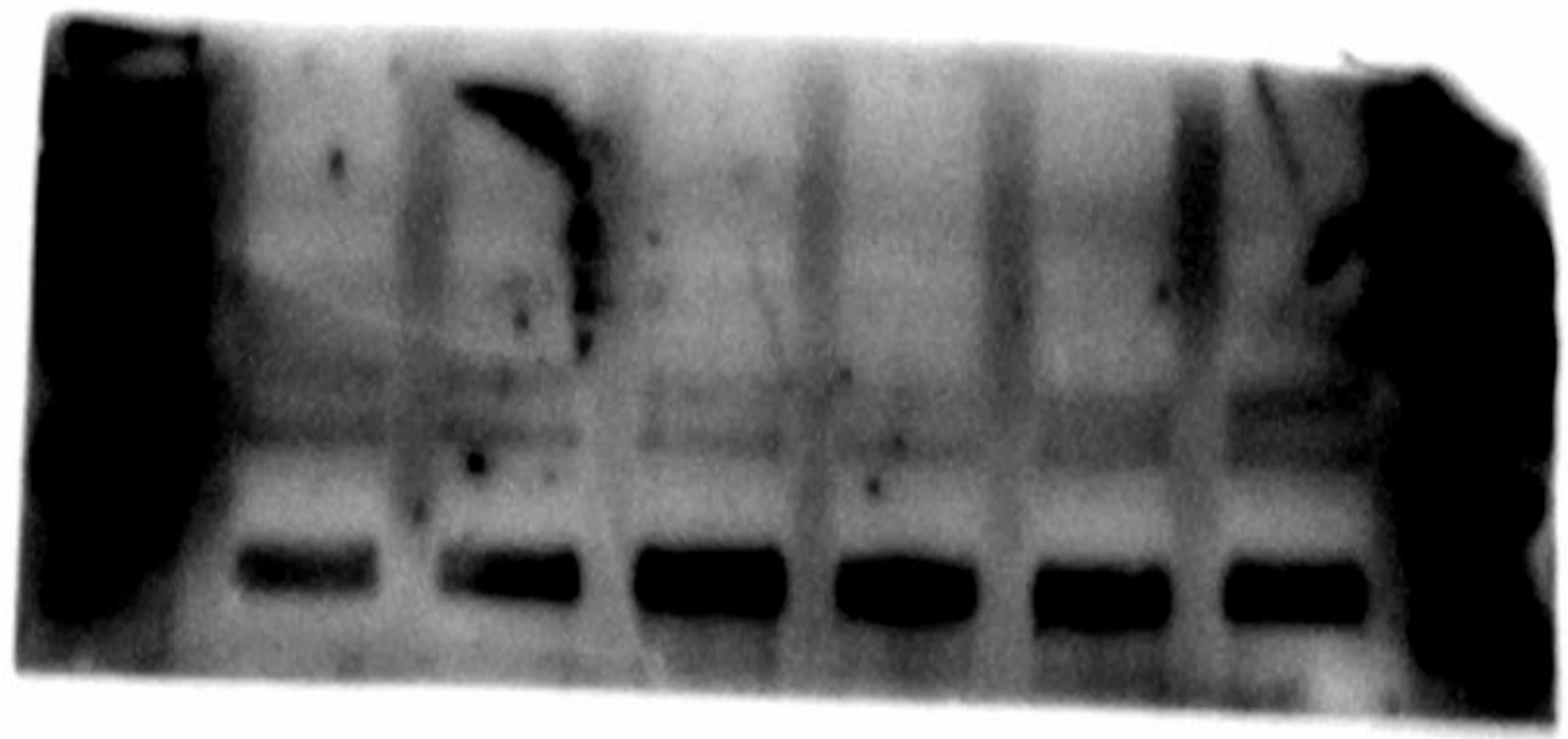

M 1 2 3 4 5 6

M= Protein Marker (kDa)

1 = Sham

2 = Sham

3 = SAE

4 = SAE

5 = SAE + $NaHCO_3$

6 = SAE + $NaHCO_3$

Raw image for Figure 7B (β-Actin internal control)

~187(kDa)

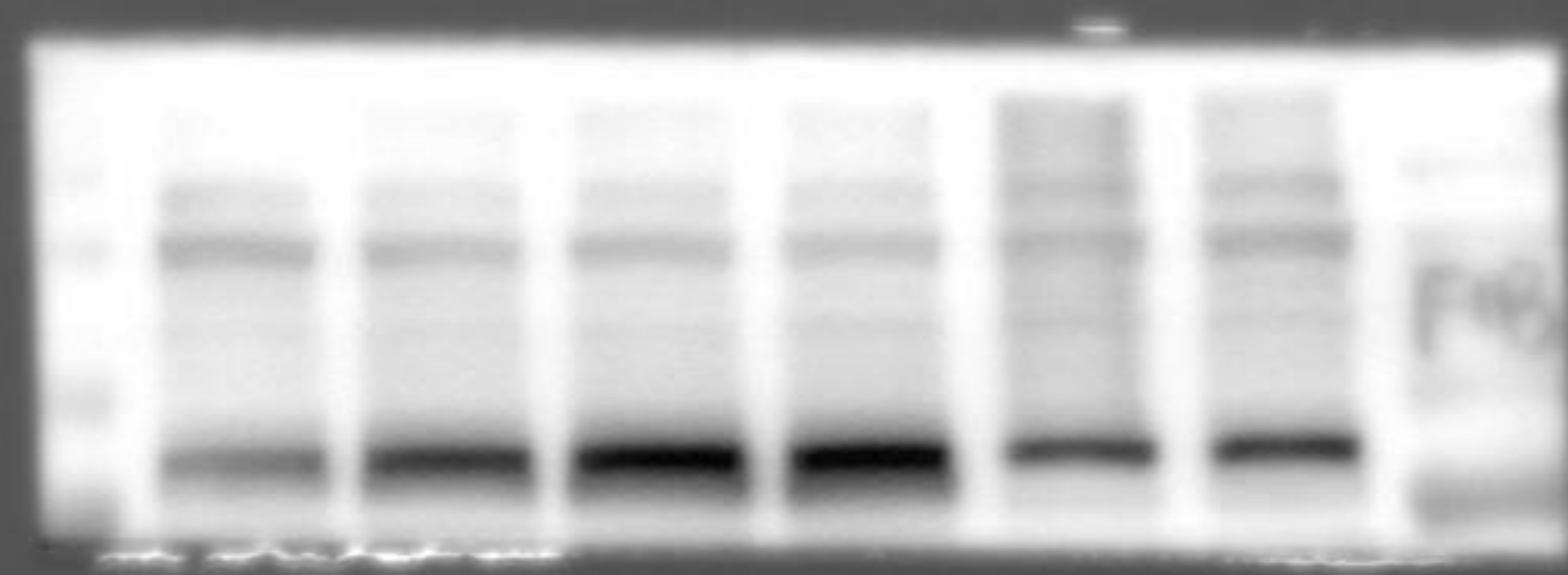

M 1 2 3 4 5 6

M= Protein Marker (kDa)

1 = Sham

2 = Sham

3 = SAE

4 = SAE

5 = SAE + $NaHCO_3$

6 = SAE + $NaHCO_3$
